# Preliminary analysis of *Psoroptes ovis* transcriptome in different developmental stages

**DOI:** 10.1186/s13071-016-1856-z

**Published:** 2016-11-04

**Authors:** Man-Li He, Jing Xu, Ran He, Neng-Xing Shen, Xiao-Bin Gu, Xue-Rong Peng, Guang-You Yang

**Affiliations:** 1 Department of Parasitology, College of Veterinary Medicine, Sichuan Agricultural University, Chengdu, 611130 China; 2 Department of Chemistry, College of Life and Basic Science, Sichuan Agricultural University, Chengdu, 611130 China

**Keywords:** *Psoroptes ovis*, Transcriptome, Differentially expressed genes (DEGs), RNA-Seq

## Abstract

**Background:**

Psoroptic mange is a chronic, refractory, contagious and infectious disease mainly caused by the mange mite *Psoroptes ovis*, which can infect horses, sheep, buffaloes, rabbits, other domestic animals, deer, wild camels, foxes, minks, lemurs, alpacas, elks and other wild animals. Features of the disease include intense pruritus and dermatitis, depilation and hyperkeratosis, which ultimately result in emaciation or death caused by secondary bacterial infections. The infestation is usually transmitted by close contact between animals. Psoroptic mange is widespread in the world. In this paper, the transcriptome of *P. ovis* is described following sequencing and analysis of transcripts from samples of larvae (i.e. the Pso_L group) and nymphs and adults (i.e. the Pso_N_A group). The study describes differentially expressed genes (DEGs) and genes encoding allergens, which help understanding the biology of *P. ovis* and lay foundations for the development of vaccine antigens and drug target screening.

**Methods:**

The transcriptome of *P. ovis* was assembled and analyzed using bioinformatic tools. The unigenes of *P. ovis* from each developmental stage and the unigenes differentially between developmental stages were compared with allergen protein sequences contained in the allergen database website to predict potential allergens.

**Results:**

We identified 38,836 unigenes, whose mean length was 825 bp. On the basis of sequence similarity with seven databases, a total of 17,366 unigenes were annotated. A total of 1,316 DEGs were identified, including 496 upregulated and 820 downregulated in the Pso_L group compared with the Pso_N_A group. We predicted 205 allergens genes in the two developmental stages similar to genes from other mites and ticks, of these, 14 were among the upregulated DEGs and 26 among the downregulated DEGs.

**Conclusion:**

This study provides a reference transcriptome of *P. ovis* in absence of a reference genome. The analysis of DEGs and putative allergen genes may lay the foundation for studies of functional genomics, immunity and gene expression profiles of this parasitic mite species.

**Electronic supplementary material:**

The online version of this article (doi:10.1186/s13071-016-1856-z) contains supplementary material, which is available to authorized users.

## Background

Psoroptic mange is a chronic, contagious and infectious disease caused by the mange mite *Psoroptes ovis*, which infects horses, sheep, buffaloes, rabbits, other domestic animals, deer, wild camels, foxes, minks, lemurs [1, 2], alpacas [3, 4], elks [5, 6] and other wild animals*. Psoroptes ovis* mites are divided into different subspecies or variants according to their host species, for example, *P. ovis* var. *cuniculi* (host: rabbit), *P. ovis* var. *equi* (host: horse), *P. ovis* var. *cervinus* (host: deer), *P. ovis* var. *natalensis* (host: buffalo), etc. [7]. The sophisticated interaction between the host and the mite results into mite infestation [8], which is characterized by skin lesions and formation of characteristic yellowish scabs [9, 10].

*Psoroptes ovis* is a non-burrowing, surface secretions feeder, capable of consuming a variety of body fluids, lymph and red blood cells [11]; the mites do not appear to penetrate beyond the stratum corneum and survive on the epidermis of mammals [12]. Mites abrade the cuticle and deposit allergens when establishing themselves on the host, that results in severe inflammatory responses [13, 14] and the production of an exudate that, in turn, represents a food source for the mites [15]. Features of psoroptic mange include intense pruritus, extensive dermatitis, depilation and hyperkeratosis, which ultimately result in emaciation or death caused by secondary bacterial infections. The infestation is usually transmitted by close contact between animals [9].

Psoroptic mange is widespread in the world. In agriculture, *P. ovis* infestations can cause lower feed conversion efficiency, poor weight gain, poor quality of leather and reduced carcass traits [9, 16–18]. Current methods of prevention and control rely heavily on chemotherapy; however, there are concerns with this approach that incluse parasite resistance to chemoterapeutics, biological residues and toxic effects on the environment. Consequently, it is necessary to develop novel strategies for mite prevention and control [19]. One approach is the combination of promoting animal resistance to the infection and exploring new acaricidal drugs, via the discovery of novel drug targets. Alternative control strategies may also rely on vaccine development. However, a lack of available sequence information hinders progress in these areas. This far, only 1,545 unique *P. ovis* expressed sequence tags (ESTs) have been identified [20]; there is no available reference genome.

Since Velculescu et al. [21] first described the transcriptome of yeast in 1997, transcriptome research has become a hot topic in biology. With second generation massively parallel sequencing platforms, transcriptome sequencing (RNA-Seq) has become widely used. In terms of parasites, the transcriptomes of *Plasmodium falciparum* [22], *Trypanosoma brucei* [23], *Schistosoma mansoni* [24], *Trichuris suis* [25], *Dermanyssus gallinae* [26] and others [27–29] have been sequenced. To better understand the intricacy of gene function and the activity of cells, description of the transcriptome is useful [30]. In this paper, RNA-Seq techniques were applied to the study of the transcriptome of *P. ovis*, to assist current understanding of the biology of this mite and to lay foundations for vaccine antigen development and drug target screening.

## Methods

### *P. ovis* var. *cuniculi* collection

*Psoroptes ovis* var. *cuniculi* were reared on rabbits at the Laboratory Animal Center of Sichuan Agricultural University, China. Scrapings in the external auditory canal were harvested using tweezers, placed in microliter plates, and incubated at 37 °C for 2 h [31]. *P. ovis* that emerged from the scrapings were divided into two groups: larvae (named the Pso_L group), and nymphs and adults (named the Pso_N_A group). The Pso_L group was composed of 100 *P. ovis* larvae, and the Pso_N_A group was composed of 60 *P. ovis* nymphs and adults. Care was taken to remove contaminating skin debris by thorough washing. Larvae (160 mg) and nymphs and adults (170 mg) were preserved immediately at −80 °C after harvesting.

### Library preparation for transcriptome sequencing

Sequencing was carried out on a second generation sequencing platform (RNA-Seq) [32]. Total RNA was extracted with Trizol reagent (Invitrogen, Carlsbad, USA) from the two groups of *P. ovis* var. *cuniculi* in accordance with the manufacturer’s recommendations. A total of 35 μg RNA from the Pso_L group and 41.76 μg RNA from the Pso_N_A group were used as input materials for RNA sample preparation. The RNA integrity number (RIN) values of both samples were > 7.0. Sequencing libraries were obtained using the Illumina TruSeq™ RNA Sample Preparation Kits (Illumina, San Diego, USA) according to the manufacturer’s instructions, and index codes were added to link sequences to each sample. For detailed steps, please refer to Wu et al. [28]. Finally, PCR products were purified using the AMPure XP system (Beckman Coulter, Beverly, USA) and the qualities of the sequencing libraries were assessed on the Agilent Bioanalyzer 2100 system (Agilent Technologies, CA, USA). The library preparations were sequenced on an Illumina HiSeq 2000 platform (Illumina,USA). RNA-Seq data were produced by Beijing Novegene Bioinformatics Technology Co., Ltd.

### Bioinformatic analysis

Before assembly, clean reads (clean data) were obtained by removing reads including adapters, reads including poly-N, and low quality reads including more than 10 % bases with q-value ≤ 20 from the raw data. Q20, Q30, GC-content and sequence duplication level of the clean data were calculated. All downstream analyses were performed using clean reads. The Trinity program [33] (http://trinityrnaseq.sourceforge.net/) was used for *de novo* assembly of the sequence data from *P. ovis* var. *cuniculi.* Finally, non-redundant unigenes could be identified until they could not be further elongated. For details of the data processing steps, please refer to Wu et al. [28].

Blastx alignment was carried out between unigenes and databases such as NR (NCBI non-redundant protein sequences), NT (NCBI nucleotide sequences), KO (KEGG Orthology), SwissProt (a manually annotated and peer-reviewed protein sequence database), PFAM (Protein Family), GO (Gene Ontology) and KOG (the euKaryotic Ortholog Groups database). Bioinformatics analyses were conducted as previously described [27]. In this analysis, the Blast2GO program [34] and WEGO software [35] were applied. Coding sequences (CDS) were predicted by comparison with sequences from other eukaryotes. The direction and CDS of unigenes in databases were obtained based on the best alignment results. Unigenes that could not be aligned to the above databases were scanned using the ESTScan software [36] to obtain the CDS and the sequence direction.

### Analysis of gene expression

The Bowtie software was used for mapping clean reads to the assembled transcriptome. The percentage gene expression coverage was estimated by counting the reads numbers mapped to each gene. Expression levels of individual unigenes were analysed, and differences in gene expression levels were compared between samples from different life-cycle stages of the mite. The standard method of RPKM (reads per kb per million reads) [37] quantify gene expression. The input data for differential gene expression is the read count data obtained in the analysis of gene expression level. Firstly, the read count data was standardized by the edgeR program package through one scaling normalized factor [38]. Secondly, the DEGseq R package (1.12.0) was used for analysis of differential gene expression; the *P* values were adjusted using the Benjamini and Hochberg method; the screening threshold was *Q* value < 0.005 and |log_2_fold-change| > 1 [39]. Blastx alignment was carried out between differential expression genes and KEGG database (Kyoto encyclopedia of genes and genomes) [40]. KOBAS software (2.0) was used to test the statistical enrichment of differential expression genes in KEGG pathways; the statistic method was hypergeometric test; the *P*-values were adjusted using the Benjamini and Hochberg method; corrected *P*-value < 0.05 was set as the threshold for significant enrichment pathway [41].

### Real-time PCR (qRT-PCR) validation

To validate the *P. ovis* expression data, qRT-PCR was employed to analyze differentially expressed genes (DEGs). Primer pairs for 9 differentially expressed genes and the housekeeping gene *GAPDH-like* are available (Additional file 1: Table S1). For qRT- PCR, an ABI7500 FAST real-time PCR System (Applied Biosystems, Forster, USA) and a SYBR®Premix Ex *Taq™* II Kit (Takara, Japan) were applied in accordance to the manufacturers’ recommendations. The qRT-PCR conditions were 95 °C for 2 min, followed by 40 cycles of 94 °C for 20 s and 58 °C for 20 s. The final melting curve was analyzed. The relative expression level of each gene was calculated using the 2^-△△Ct^ method [42].

### Prediction of putative allergens

To predict putative allergens, the unigenes of *P. ovis* in each developmental stage and the differentially expressed unigenes of *P. ovis* were compared by Blast against allergen protein sequences from the allergen database website (http://www.allergome.org).

## Results

### Illumina sequencing and assembly of *P. ovis* transcriptome data

**Fig. 1:**
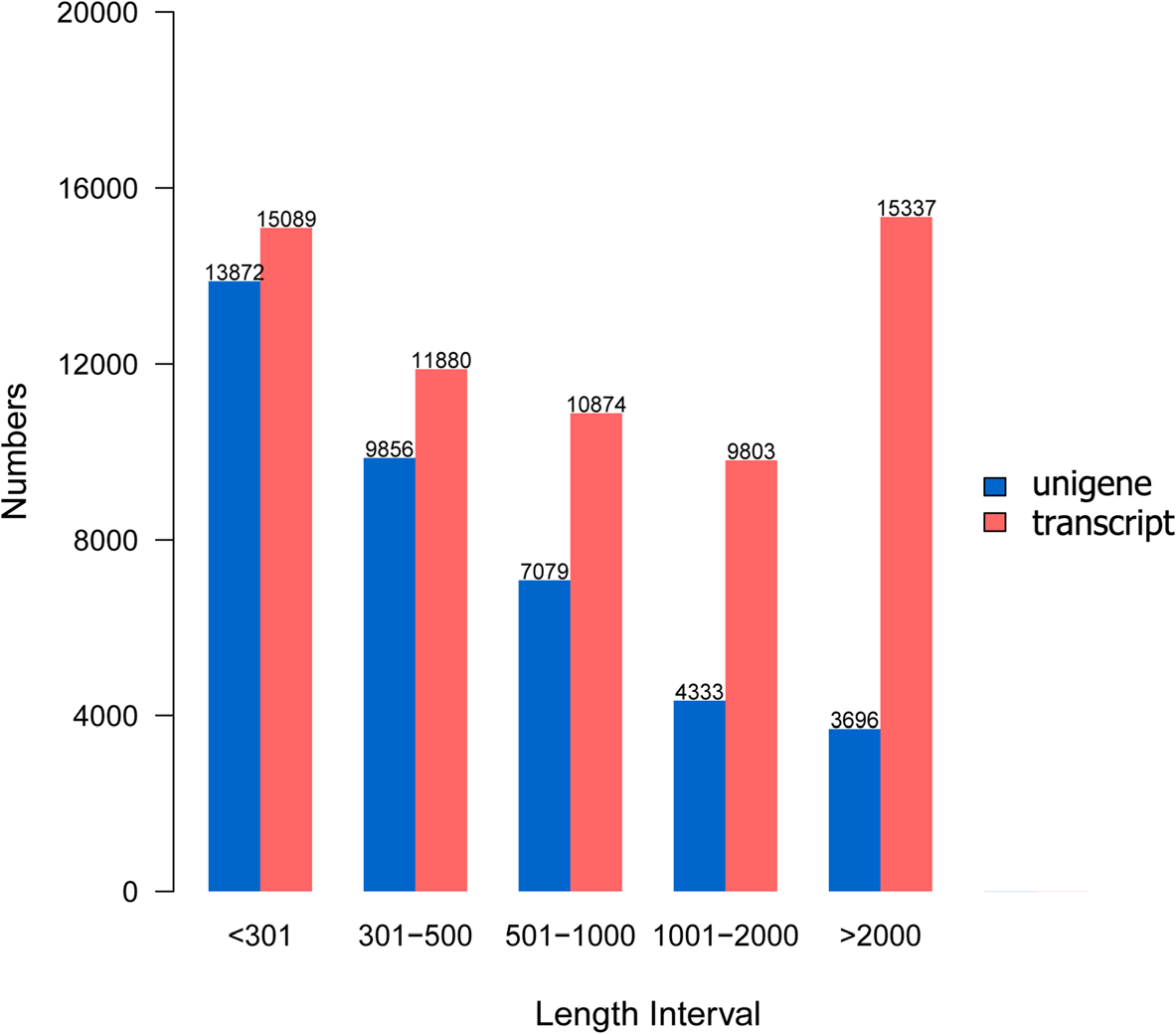
Length distribution of *P. ovis* transcripts and unigenes

We obtained 59.4 million and 55.4 million raw reads from the Pso_L and Pso_N_A groups, respectively, by RNA-Seq. The raw sequence data has been submitted to the SRA division of GenBank (project accession no.: PRJNA317241). After a rigorous screening process, 56.6 million clean reads from the Pso_L group with a Q20 of 96.91 % and a GC of 35.85 % were retained. There were 52.9 million clean reads from the Pso_N_A group with a Q20 of 96.54 % and a GC of 34.90 % (Additional file 2: Table S2). Using the Trinity software, all clean data were assembled into a transcriptome, which was used as a reference sequence for further analyses. We obtained 62,983 transcripts with an average length of 1,480 base pairs (bp) and an N50 of 3,208 bp. Transcripts were clustered using the TGICL program; 38,836 unigenes were generated. The mean length and N50 were 825 bp and 1,607 bp, respectively. Of the 38,836 unigenes, 15,108 (38.90 %) were ≥ 500 bp, and 8,029 (20.67 %) were ≥ 1,000 bp (Additional file 3: Table S3 and Additional file 4: Table S4). The length distribution of the transcripts and unigenes is shown in Fig. 1.

### Functional annotation of unigenes in *P. ovis*

**Fig. 2:**
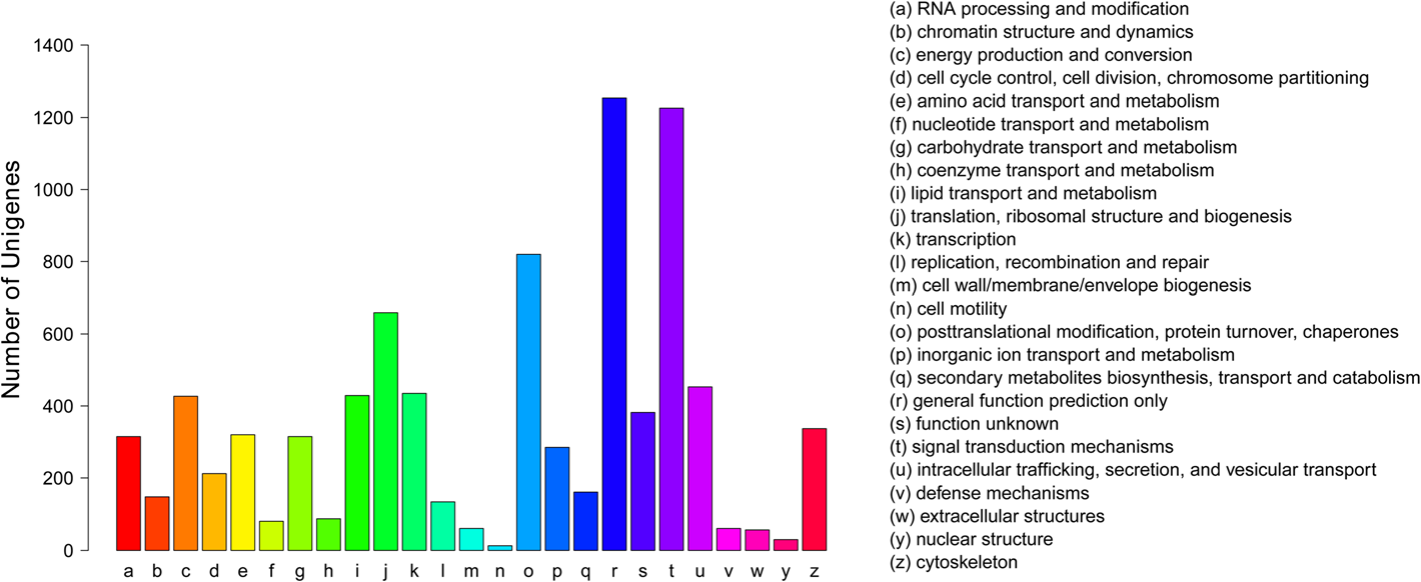
Histogram presenting clusters of orthologous groups (KOG) classifications. Of 38,836 unigenes, 7,798 sequences were assigned to 25 KOG classifications

The unigenes of the *P. ovis* transcriptome were compared with sequences in the NR, NT, KO, SwissProt, PFAM, GO and KOG databases using the Blastx program. Many of the unigenes showed homology when mapped against the databases (Table 1). When aligned through the KOG database, 7,798 unigenes were categorized into 25 molecular families (Fig. 2). Only 16.07 % (1,253/7,798) of the unigenes were contained in the cluster ‘general function prediction only’. ‘Signal transduction mechanisms’, ‘post-translational modification, ‘protein turnover, chaperones’, ‘translation, ribosomal structure and biogenesis’ and ‘intracellular trafficking, secretion, and vesicular transport’ included 15.72 % (1,226), 10.52 % (820), 8.45 % (659) and 5.81 % (453) of the unigenes, respectively. ‘Function unknown’ contained 4.90 % (382) of the unigenes.

**Table 1:** Annotations of the *P. ovis* transcriptome. The percentage, in a database, equals the number of unigenes that were annotated successfully accounted for the proportion of the total number of unigenes

	Number of unigenes	Percentage (%)
Annotated in NR	12,121	31.21
Annotated in NT	7,681	19.77
Annotated in KO	6,624	17.05
Annotated in SwissProt	10,805	27.82
Annotated in PFAM	12,157	31.3
Annotated in GO	13,213	34.02
Annotated in KOG	7,798	20.07
Annotated in all databases	2,330	5.99
Annotated in at least one database	17,366	44.71
Total unigenes	38,836	

*Abbreviations*: *NR* NCBI non-redundant protein sequences, *NT* NCBI nucleotide sequences, *KO* KEGG orthology, *SwissProt* a manually annotated and reviewed protein sequence database, *PFAM* protein family, *GO* gene ontology, *KOG* the euKaryotic Ortholog Groups database

**Fig. 3:**
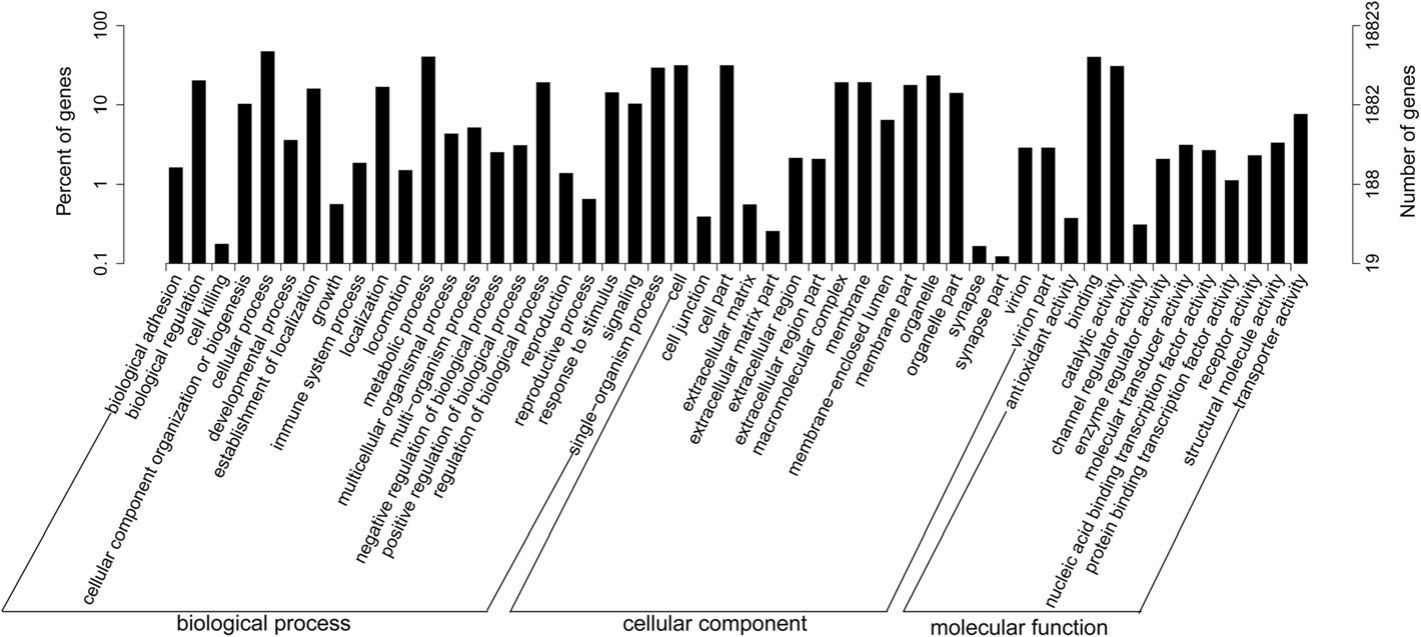
GO annotations of unigenes in the *P. ovis* transcriptome

GO functional cluster analysis was also undertaken. Of the most significant Blastx hits against the NR known species dataset, 13,213 unigenes were mapped to GO terms using the Blast2GO program (Table 1). These GO terms were categorized into the 3 main GO categories and 55 subcategories. The 3 main GO categories (i.e. molecular function, cellular components and biological process) contain 14, 18 and 23 subcategories, respectively (Fig. 3). The predominant GO annotation was ‘biological processes’ (46,840, 48.26 % of the total), followed by ‘cellular component’ (32,627, 33.61 %) and ‘molecular function’ (17,594, 18.13 %).

### KEGG pathway analysis of unigene consensus sequences

**Fig. 4:**
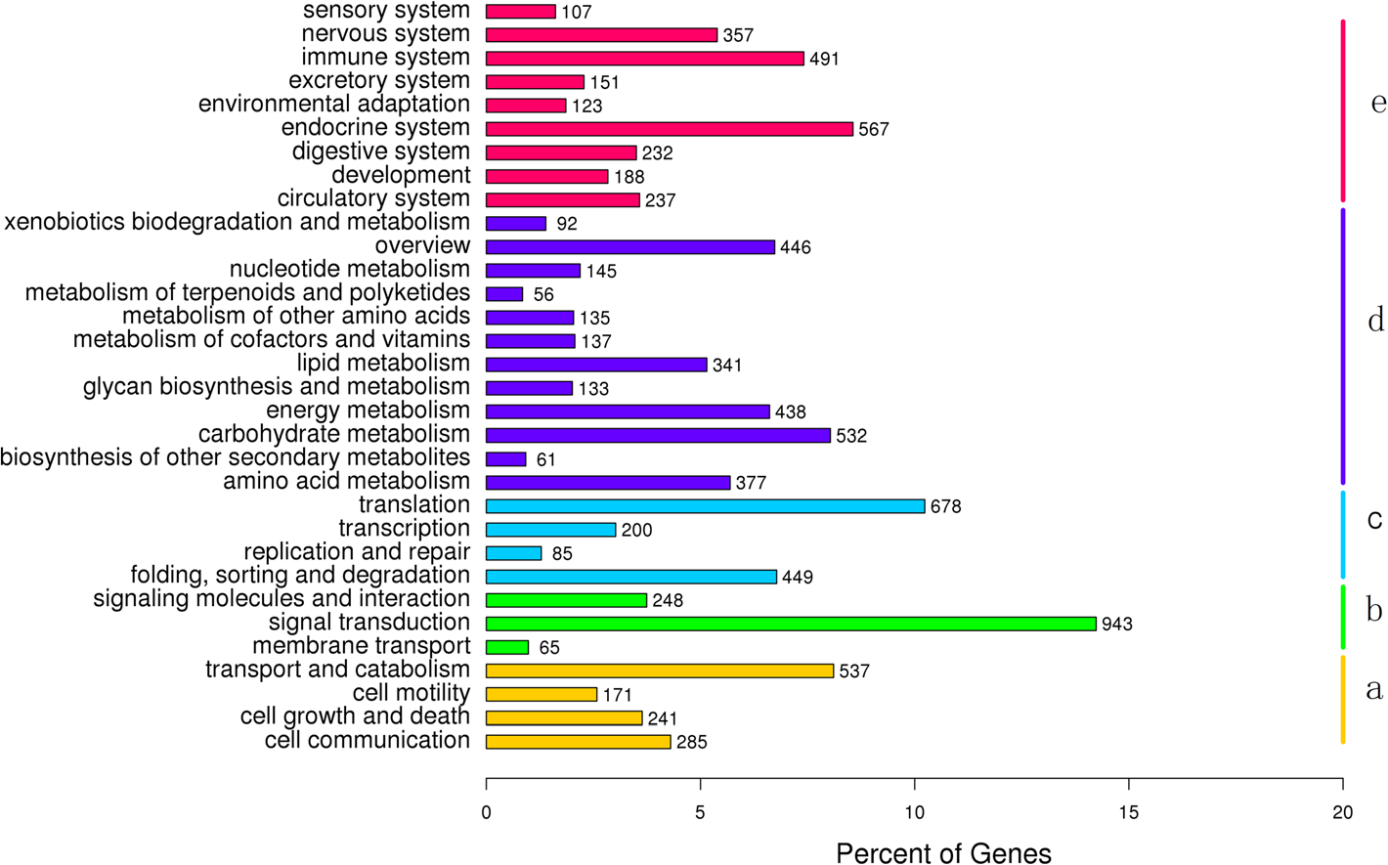
KEGG categories of *P. ovis* unigenes. Overall, 6,624 unigenes were annotated against the KEGG database. **a** Cellular processes; **b** Environmental information processing; **c** Genetic information processing; **d** Metabolism; **e** Organismal systems

We were able to assign 2,201 different KO terms and 265 KEGG pathways to the *P. ovis* transciptome to 6,624 unigenes (Additional file 5: Table S5). The KEGG pathways included six categories (‘Metabolism’, ‘Genetic Information Processing (GIP)’, ‘Environmental Information Processing (EIP)’, ‘Cellular Processes’, ‘Organismal Systems’, and ‘Human Diseases’). We removed all the pathways belonging to Human Diseases and showed the remaining classification results in a histogram (Fig. 4). The most abundant subcategory in the ‘EIP’ category was ‘signal transduction’ (943 unigenes, 14.24 %); the most abundant subcategory in the ‘GIP’ category was ‘translation’ (678, 10.24 %); and the most abundant subcategory in the ‘Organismal Systems’ was ‘endocrine system’ (567, 8.56 %). Among the 265 KEGG pathways, the predominant terms were: ‘ribosome’ (ko03010, 393), ‘carbon metabolism’ (ko01200, 287), ‘protein processing in endoplasmic reticulum’ (ko04141, 209), ‘oxidative phosphorylation’ (ko00190, 202), ‘PI3K-Akt signaling pathway’ (ko04151, 181) and ‘MAPK signaling pathway’ (ko04010, 180).

### Alignment of CDS

The CDS of unigenes were determined using the Blastx program, then by translating the corresponding *P. ovis* gene sequence into amino acids in accordance with the standard code table. The unigenes that did not match known genes were predicted using the ESTScan program. We identified 17,731 (45.66 %) CDS from the 38,836 unigenes in the *P. ovis* transcriptome. Of these, 12,950 were identified using the Blastx algorithmic program; their length distribution is shown in Additional file 6: Figure S1; the ESTScan software identified 4,781 CDS and their length distribution is shown in Additional file 7: Figure S2.

### Analysis of differentially expressed genes from *P. ovis* in different developmental stages

To detect gene expression differences in different developmental life-cycle stages of *P. ovis*, we analyzed differentially expressed genes in the Pso_L (larval) and Pso_N_A (nymph/adult) groups. A total of 1,316 DEGs (Additional file 8: Table S6) were identified, including 496 upregulated unigenes and 820 downregulated unigenes in the Pso_L group compared with the Pso_N_A group. DEGs were assigned to 151 KEGG pathways. Amongst them, ‘lysosome’ (ko04142, 31 unigenes) and ‘phagosome’ (ko04145, 29) showed significant enrichment (Additional file 9: Table S7). The upregulated DEGs in the Pso_L group were assigned to 101 KEGG pathways (Additional file 10: Table S8). Amongst them, 5 showed significant enrichment, including ‘NF-kappa B signaling pathway’ (ko04064, 11), ‘cytokine-cytokine receptor interaction’ (ko04060, 12), ‘TNF signaling pathway’ (ko04668, 11), ‘phagosome’ (ko04145, 16)’, and ‘regulation of actin cytoskeleton’ (ko04810, 15). Downregulated DEGs in the Pso_L group relative to Pso_N_A were assigned to 138 KEGG pathways (Additional file 11: Table S9). Amongst them, 3 showed significant enrichment, including ‘lysosome’ (ko04142, 19), ‘protein processing in endoplasmic reticulum’ (ko04141, 24), and ‘cell cycle’ (ko04110, 14).

**Fig. 5:**
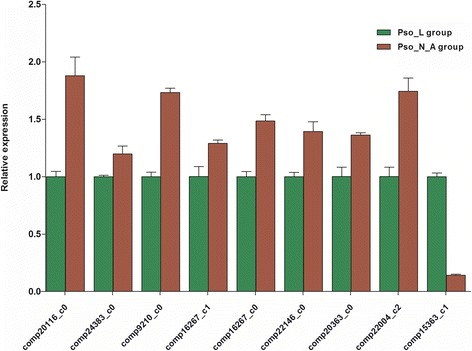
qRT-PCR validation of nine selected genes that were differentially expressed between the Pso_L group (samples from *P. ovis* larvae) and the Pso_N_A group (samples from adults and nymphs) in the transcriptomic data. Error bars indicate SDs

qRT-PCR was employed to validate the *P. ovis* transcriptome data. Specifically, nine DEGs were selected for experimental validation and statistical analysis. *Pdis* (comp20116_c0), *bcap31* (comp24383_c0), *sec61a* (comp9210_c0), *hsp90* (comp16267_c1, comp16267_c0, comp22146_c0), *p97* (comp20363_c0), and *rad32* (comp22004_c2) were found to be upregulated in the Pso_N_A group relative to the Pso_L group by qRT-PCR. *hsp70* (comp15363_c1) was downregulated (Fig. 5). These results were consistent with the transcriptome sequencing data.

### Protein processing in the endoplasmic reticulum

To help obtain a better understanding of the biochemistry and physiology of *P. ovis*, we chose the ‘protein processing in the endoplasmic reticulum’ pathway of ‘GIP’ (Additional file 12: Figure S3) as a case-study for further analysis. Across the transcriptome, this pathway was mapped to 209 unigenes grouped into three categories (‘endoplasmic reticulum protein processing’, ‘endoplasmic reticulum stress response’ and ‘endoplasmic reticulum associated degradation-ERAD’). This pathway was one of the most enriched in upregulated DEGs in the Pso_N_A group relative to the Pso_L group; there were 24 upregulated DEGs related to this pathway. ERAD, coupled with the ubiquitin-proteasome degradation pathway [43], is the main pathway of intracellular protein degradation and is involved in > 80 % of protein degradation in the cell. Five key genes in ERAD, heat-shock protein *hsp90*, heat-shock protein *hsp70*, transitional endoplasmic reticulum ATPase *p97*, ERAD enhancers mannosidase *edem*, and transporters *sec61,* were mapped by 14, 19, 8, 3 and 3 transcripts, respectively.

### Prediction of allergen genes

The unigenes of *P. ovis* in the two developmental stages were compared by Blastx against allergen protein sequences, and 1,294 putative allergen genes were identified (Additional file 13: Table S10). Amongst them, there were 205 which resulted in Blast hits to genes from other mites (such as *Dermatophagoides pteronyssinus*, *Dermatophagoides farina*, etc.) and ticks (Additional file 14: Table S11), and 1,089 which did not result in Blast hits to genes from other mites or ticks (Additional file 15: Table S12). The upregulated DEGs were similarly analyzed in the Pso_L group relative to the Pso_N_A group, and 72 putative allergen genes were predicted (Additional file 16: Table S13). Of these, 14 were shared with other mites and ticks (Table 2), and 58 were not (Additional file 17: Table S14). A similar analysis of the downregulated DEGs predicted 116 putative allergen genes (Additional file 18: Table S15), of which 26 produced Blast hits to sequences from mites and ticks (Table 3), while 90 did not (Additional file 19: Table S16).

**Table 2:** Allergen genes which Blast hits with genes from mites and ticks among the upregulated DEGs

Unigene ID	Allergen protein ID	Species	Description	Blast e-value
comp21772_c1	A0A088SAG5	*Dermatophagoides farinae*	A0A088SAG5_DERFA Der f 30 allergen	1.12e^-102^
comp16294_c0	L7UZ91	*Dermatophagoides farinae*	L7UZ91_DERFA Ferritin	1.48e^-66^
comp22600_c16	C6ZDB5	*Tyrophagus putrescentiae*	C6ZDB5_TYRPU Tyr p 3	1.02e^-11^
comp21796_c0	Q8WQ47	*Lepidoglyphus destructor*	TBA_LEPDS Tubulin alpha chain	0
comp20560_c0	A0A088SAY1	*Dermatophagoides farinae*	A0A088SAY1_DERFA Der f 31 allergen	4.63e^-06^
comp7410_c0	A0A088SAY1	*Dermatophagoides farinae*	A0A088SAY1_DERFA Der f 31 allergen	1.70e^-11^
comp22325_c14	L7N6F8	*Dermatophagoides pteronyssinus*	L7N6F8_DERPT Dust mite allergen	5.95e^-06^
comp21866_c0	Q4JK69	*Dermatophagoides pteronyssinus*	Q4JK69_DERPT Group 15 allergen protein short isoform	8.53e^-76^
comp22602_c8	Q6Y2F9	*Dermatophagoides pteronyssinus*	Q6Y2F9_DERPT HDM allergen	9.29e^-18^
comp15615_c0	A0A088SAX2	*Dermatophagoides farinae*	A0A088SAX2_DERFA Triosephosphate isomerase	2.91e^-120^
comp15363_c1	A0A088SAS1	*Dermatophagoides farinae*	A0A088SAS1_DERFA Der f 28 allergen	4.99e^-61^
comp22460_c6	Q6Y2F9	*Dermatophagoides pteronyssinus*	Q6Y2F9_DERPT HDM allergen	9.80e^-27^
comp35289_c0	A0A088SAG5	*Dermatophagoides farinae*	A0A088SAG5_DERFA Der f 30 allergen	1.16e^-31^
comp22588_c0	L7UZ85	*Dermatophagoides farinae*	L7UZ85_DERFA Alpha–actinin	4.46e^-07^

**Table 3:** Allergen genes which Blast hits with genes from mites and ticks among the downregulated DEGs

Unigene ID	Allergen protein ID	Species	Description	Blast e-value
comp22562_c0	A0A088SCP3	*Dermatophagoides farinae*	A0A088SCP3_DERFA Der f 32 allergen	5.57e^-179^
comp22544_c0	Q8N0N0	*Dermatophagoides pteronyssinus*	Q8N0N0_DERPT Group 14 allergen protein (Fragment)	1.33e^-20^
comp16295_c1	A1KXH2	*Dermatophagoides farinae*	A1KXH2_DERFA Der f 1 allergen	2.58e^-31^
comp19324_c1	Q9U6R7	*Dermatophagoides farinae*	Q9U6R7_DERFA 98 kDa HDM allergen	7.44e^-94^
comp16185_c0	Q8WQ47	*Lepidoglyphus destructor*	TBA_LEPDS Tubulin alpha chain	2.44e^-91^
comp20889_c0	L7N6F8	*Dermatophagoides pteronyssinus*	L7N6F8_DERPT Dust mite allergen	2.81e^-07^
comp7240_c0	Q9Y197	*Dermatophagoides pteronyssinus*	Q9Y197_DERPT Alpha–amylase (Fragment)	0
comp19263_c0	L7N6F8	*Dermatophagoides pteronyssinus*	L7N6F8_DERPT Dust mite allergen	2.67e^-07^
comp22789_c0	Q1EIQ3	*Psoroptes ovis*	PEPT1_PSOOV Peptidase 1	0
comp22115_c5	A1KXG7	*Dermatophagoides farinae*	A1KXG7_DERFA Der f 7 allergen	1.87e^-59^
comp21147_c0	Q9U1G2	*Lepidoglyphus destructor*	ALL7_LEPDS Mite allergen Lep d 7	7.02e^-21^
comp22817_c0	E0A8N8	*Dermatophagoides pteronyssinus*	E0A8N8_DERPT Der p 13 allergen	9.01e^-80^
comp21324_c0	P49273	*Dermatophagoides pteronyssinus*	ALL7_DERPT Mite allergen Der p 7	1.19e^-93^
comp22866_c0	A8B8G7	*Dermatophagoides farinae*	A8B8G7_DERFA Group 21 allergen	3.91e^-48^
comp16295_c0	Q1EIQ3	*Psoroptes ovis*	PEPT1_PSOOV Peptidase 1	6.01e^-80^
comp16271_c0	L7UZ91	*Dermatophagoides farinae*	L7UZ91_DERFA Ferritin	7.49e^-56^
comp7938_c0	Q965E2	*Psoroptes ovis*	ALL2_PSOOV Mite group 2 allergen Pso o 2	2.18e^-73^
comp16261_c0	Q52PV9	*Tyrophagus putrescentiae*	Q52PV9_TYRPU Alpha–tubulin	1.88e^-17^
comp22807_c0	Q8N0N0	*Dermatophagoides pteronyssinus*	Q8N0N0_DERPT Group 14 allergen protein (Fragment)	0
comp22811_c0	L7N6F8	*Dermatophagoides pteronyssinus*	L7N6F8_DERPT Dust mite allergen	6.09e^-13^
comp23114_c0	A1KXH1	*Dermatophagoides farinae*	A1KXH1_DERFA Der f 13 allergen	2.73e^-37^
comp22853_c0	A8B8I1	*Dermatophagoides farinae*	A8B8I1_DERFA Der f 5 allergen	2.16e^-45^
comp7750_c0	L7UW58	*Dermatophagoides farinae*	L7UW58_DERFA Translation elongation factor 2 (Fragment)	0
comp22673_c2	Q86R84	*Dermatophagoides farinae*	Q86R84_DERFA 60 kDa allergen Der f 18p	0
comp16261_c1	Q8WQ47	*Lepidoglyphus destructor*	TBA_LEPDS Tubulin alpha chain	3.93e^-87^
comp22539_c0	A3F5F1	*Dermatophagoides farinae*	A3F5F1_DERFA Der f 2 allergen	2.97e^-14^

## Discussion

### Coverage and quality of consensus sequences

Analysis of transcriptome data is useful to the understanding of genomic function and cellular activities in organisms [30]. Paired-end transcriptome sequencing technology is an effective approach for the analysis of transcriptomic data [44], and is also efficient in elucidating the complexity of the transcriptome. In the present study, the transcriptome of *P. ovis* was determined using a HiSeq 2000 paired-end sequencing platform and Trinity assembling software. This analysis provided extensive coverage of the transcriptome in long fragments, which provided us with the opportunity to: (i) analyze gene expression; (ii) predict new genes; and (iii) explore metabolic pathways in the absence of a reference genome.

Kenyon et al. sequenced 484 *P. ovis* ESTs in 2003 [45]. To further expand knowledge of the *P. ovis* transcriptome, a cDNA library of *P. ovis* was constructed by Burgess et al. [20]; 1,574 ESTs were sequenced and, combined with 484 previously obtained *P. ovis* sequences such that, resulted in 1,545 mite sequences. Li et al. sequenced 33 *P. ovis* var. *cuniculi* ESTs by constructing a cDNA library [46]. Thus, overall, relatively few *P. ovis* sequences were obtained in previous investigations. In the present study, reads were assembled into 38,836 unigenes. On the basis of sequence similarity sequences stored in seven databases (NR, NT, KO, SwissProt, PFAM, GO and KOG), 17,366 unigenes were annotated, which greatly expands the *P. ovis* transcript database. The average length of *P. ovis* unigenes was 825 bp, which is longer than some species in earlier studies, for example, *Taenia pisiformis* (398 bp) [27]. The unigene annotations included a description of the gene or protein name, prediction of conserved regions and analysis of GO terms and metabolic/signaling pathways (only in the KEGG database), which provides biological function information, metabolic/signaling pathways of nominee genes at a specific time. Such data enables a more in-depth understanding of gene expression in *P. ovis*. In this study, 17,260 CDS were obtained. Amongst them, we identified many novel genes, which provides a platform for research into *P. ovis* functional genes. For example, heat-shock proteins [47] can be screened as candidate diagnostic antigens.

### Gene functional clusters of the *P. ovis* transcriptome

Identified unigenes could be annotated with a variety of KOG functional clusters, which showed that transcriptomic data on *P. ovis* contained extensive transcript diversity. KOG, short for euKaryotic Ortholog Groups, is an NCBI database based on relationships between orthologous genes. Based on KOG and evolutionary relationships, homologous genes from different species can be divided into different ortholog clusters. To date, there are 4,852 KOG classifications. Orthologous genes are annotated with the same function, so functional annotation is inherited directly by other members of the same KOG cluster. In 25 molecular families, the clusters for ‘coenzyme transport and metabolism’, ‘replication, recombination and repair’, ‘intracellular trafficking secretion, and vesicular transport’ and ‘defense mechanisms’ had a meaningful impact, because they were closely related to the immune and physiological functions of *P. ovis.* Analysis of gene expression levels, GO functional clusters and prediction of protein metabolic pathways, respectively, indicated the sifting of high abundance expression genes, GO functional classification of different genes and positioning of relevant metabolic pathways. The *P. ovis* transcriptome data in this work add to the available data on this parasite and will facilitate research into gene functions and antigen mechanism.

### Analysis of putative allergen genes

Proinflammatory cytokines cause skin damage; the source of these factors may be excretions/secretions containing enzymes and allergens from mites, such as cysteine proteinases, *gst, pso2* et al.[15]. In particular, we characterized putative *P. ovis* allergen genes and analyzed differences in in gene expression between larval and adult/nymph stages, which lays the foundations for research into the pathogenic properties of these two mite stages. At present, only eight allergen genes have been studied in-depth in *P. ovis*: *pso2* [48], *actin* [49], *pso1* [50], *pso10* [51], *pso11* [51], vitellogenin [52], *gst* [53], and troponin C [31]. In this study, comparisons of *P. ovis* DEGs against allergen protein sequences resulted in 188 predicted allergen genes. Amongst them, 40 matched sequences in other mites and ticks, including some important allergen genes such as *derf15* 98 kDa chitinase [54], *derp7* allergen peptides [55], and *derf18* chitinase-like chitin-binding protein [54]. Further study of these genes will help in understanding the interactions between *P. ovis* and its hosts.

### Collection of experimental materials and analysis of DEGs

The collection of suitable experimental materials lays the foundation for subsequent experiments. cDNA libraries of *P. ovis* were constructed by Kenyon et al. [45] and Burgess et al. [20], and a cDNA library of *P. ovis* var. *cuniculi* was constructed by Hu et al.[46]*.* The experimental materials used in these studies were all mixed life-cycle stages of *P. ovis*, including larvae, nymphs and adults. In the present work, specimens of *P. ovis* var. *cuniculi* were assigned to two groups, larvae (the Pso_L group) and nymphs and adults (the Pso_N_A group). Larvae are characterized by distinct features, including a smaller body, a lighter color than nymphs and adults and three pairs of legs (rather than four in nymphs and adults) [56]. Adult males (with an average length of 396 μm), male tritonymphs (414 μm) and adult females (402 μm) have very similar body lengths [57]. The mean body widths of adult males and male tritonymphs are 380 μm and 370 μm, respectively [57]. In addition, female tritonymphs and adult males are attached during mating and cannot be separated easily. Moreover, adult females is similar morphologically to female tritonymphs except for very small differences in reproductive organs [56]. Taken together, there is difficulty in identifying and separating nymphs and adults.

We analyzed DEGs in different life-cycle stages of *P. ovis* (i.e. in larvae and nymphs/adults). A total of 1,316 DEGs were analyzed. The analysis of differential gene expression may lay the foundation for research into the development of the reproductive system. Besides, as described above, morphologically, the difference is obvious between the samples in the Pso_L and Pso_N_A groups, especially the differences of legs. It is necessary to further study if there are genes that control the fourth pair of legs in the upregulated DEGs in the Pso_N_A group relative to the Pso_L group. Therefore, the analysis of differential gene expression may also lay the foundation for research into the morphological differences between the stages of *P. ovis*.

### Analysis of ER-associated degradation pathway

The function of many unigenes is closely related to signaling and metabolic pathways. In this study, we specifically characterized the endoplasmic reticulum associated degradation pathway (ERAD) of the ‘protein processing in endoplasmic reticulum’ KEGG pathway. The ERAD pathway guides ubiquitin-mediated degradation of a range of ER-associated misfolded and normal proteins and functions in protein quality control [58]. That selective degradation of normal proteins is the base for cellular regulation of many processes [59]. For example, in order to control the cell cycle, cyclins or their inhibitors are degradated. By comparison, the degradation of quality control requires recognition of damaged or misfolded proteins. *hsp90*, *hsp70* and *edem* are key genes in the ERAD pathway. When cells are stressed (such as as a consequence of infection, heat-shock or oxidative damage), heat-shock proteins are expressed; their role is to identify misfolded proteins and mark them for ubiquitin-mediated degradation. As a molecular chaperone, lumenal heat-shock protein *hsp70* plays an important role in recognition and degradation of lumenal ERAD substrates [60]. Four cytosolic *hsp70* proteins play a key role in identifying and degrading integral membrane substrates [61].

An earlier study showed that a calnexin cycle mechanism was closely related to ERAD in the endoplasmic reticulum [62]. The calnexin cycle refered to that the newly synthesized glycoproteins began to fold after combing with transmembrane protein calnexin and soluble protein calreticulin in the endoplasmic reticulum through the glucose residues. Correctly folded glycoproteins were separated from the two proteins, while the misfolded ones would recirculate and repeat the folding process. Since only those proteins that were out of the calnexin cycle could pass into the ERAD pathway, the calnexin cycle limited the rate of misfolded proteins going into the ERAD which effected the degradation of these proteins [63]. Molinari et al. [64] found that a mannosidase-1-like protein (*edem*) could adjust a misfolded glycoprotein released from the calnexin cycle and passed into the ERAD pathway. *edem* overexpression could cause a faster release of misfolded proteins from the calnexin cycle and an earlier start of ERAD. The upregulated *edem* during endoplasmic reticulum stress may promote cell recovery by clearing the calnexin cycle and by facilitating ERAD of terminally folding-incompetent polypeptides.

In our data, 14, 19 and 3 transcripts could be mapped to heat-shock protein *hsp90*, heat-shock protein *hsp70* and ERAD enhancer mannosidase *edem* of the ERAD pathway, respectively. These important genes provide opportunities to investigate vaccine antigens against *P. ovis* and drug targets in this mite.

## Conclusion

Here we have identified 38,836 unigenes from *P. ovis* by RNA-Seq and bioinformatic analyses of sequence data. A total of 17,366 unigenes were annotated and 17,260 CDS were identified. We also analyzed 1,316 DEGs in different life-cycle stages of *P. ovis*. This work will lay the foundation for studies of functional genomics, immunoregulation mechanism, vaccine antigens and gene expression profiles of this parasitic mite species.

## Additional files

Additional file 1: Table S1. Primer sequences used for qRT-PCR. (DOCX 14 kb)
Additional file 2: Table S2. The quality of transcriptome data. (XLSX 10 kb)
Additional file 3: Table S3. The frequency distribution of splice length. (XLSX 9 kb)
Additional file 4: Table S4. The distribution of splice length. (XLSX 9 kb)
Additional file 5: Table S5. Detailed KEGG pathway categories of the *P. ovis* unigenes. (XLSX 10 kb)
Additional file 6: Figure S1. Length distribution of CDS determined using the Blastx algorithm. (TIF 1334 kb)
Additional file 7: Figure S2. Length distribution of CDS determined by ESTScan software. (TIF 1342 kb)
Additional file 8: Table S6. DEGseq analysis results for DEGs. (XLSX 110 kb)
Additional file 9: Table S7. The enrichment results of DEGs in KEGG pathways. (XLS 72 kb)
Additional file 10: Table S8. The enrichment results of upregulated DEGs in KEGG pathways. (XLSX 20 kb)
Additional file 11: Table S9. The enrichment results of downregulated DEGs in KEGG pathways. (XLS 64 kb)
Additional file 12: Figure S3. The ‘Protein processing in endoplasmic reticulum’ pathway. (TIF 46 kb)
Additional file 13: Table S10. Allergen genes among the unigenes in the two developmental stages. (XLSX 83 kb)
Additional file 14: Table S11. Allergen genes which resulted in Blast hits with genes from other mites and ticks among the unigenes of *P. ovis* in the two developmental stages. (XLSX 20 kb)
Additional file 15: Table S12. Allergen genes which did not result in Blast hits with genes from other mites or ticks among the unigenes of *P. ovis* in the two developmental stages. (XLSX 64 kb)
Additional file 16: Table S13. Allergen genes among the upregulated DEGs. (XLSX 15 kb)
Additional file 17: Table S14. Allergen genes which did not result in Blast hits with genes from other mites or ticks among the upregulated DEGs. (XLSX 13 kb)
Additional file 18: Table S15. Allergen genes among the downregulated DEGs. (XLSX 16 kb)
Additional file 19: Table S16. Allergen genes which did not result in Blast hits with genes from other mites or ticks among the downregulated DEGs. (XLSX 16 kb)

## Abbreviations

DEG: Differentially expressed gene
GO: Gene ontology
KEGG: Kyoto encyclopedia of genes and genomes
KO: KEGG Orthology
KOG: The euKaryotic Ortholog Groups database
NR: NCBI non-redundant protein sequences
NT: NCBI nucleotide sequences
PFAM: Protein family
RIN: RNA integrity number.
RPKM: Reads per kb per million reads
RT-qPCR: Reverse-transcription quantitative PCR
SwissProt: A manually annotated and reviewed protein sequence database
