# Supplementary material for: Preliminary analysis of Psoroptes ovis transcriptome in different developmental stages
Source: Parasit Vectors. 2016 Nov 4;9:570. doi: 10.1186/s13071-016-1856-z (PMC5096302; doi:10.1186/s13071-016-1856-z)
Supplement: Additional file 1: Table S1. — Primer sequences used for qRT-PCR. (DOCX 14 kb) [file 13071_2016_1856_MOESM1_ESM.docx]

**Additional file 1. Table S1.** Primer sequences used for qRT-PCR

| Gene name | Primer Sequence(5’- 3’) | Size (bp) |
| --- | --- | --- |
| GAPDH-like F | TTGTGATGGGCGTGAACC | 169 |
| GAPDH-like R | GTCTTCTGGGTGGCAGTGAT |  |
| PDIs F | AATCATTGGCAAGCCGTTAT | 194 |
| PDIs R | TTGTTCACTTCCTTCGGTCA |  |
| BCAP31 F | GTTGGTTCATCGGCATTCTA | 158 |
| BCAP31 R | GATGCTTCTGATTCGGCTTT |  |
| SEC61A F | AGTTGGTGGTCTTTGTTATTATCTG | 157 |
| SEC61A R | ATCTTTAGCACTTGAGCCCGA |  |
| HtpG1 F | TGAAAATCGCAAACAAAAAAATA | 143 |
| HtpG1 R | GGGAGATGTTCAATGGCAGAT |  |
| HtpG2 F | ACGAAAATGCCTGAAGAAAAC | 177 |
| HtpG2 R | ATCAATCAACGATTCATAGCG |  |
| HtpG3 F | GAAATGGACAAAGATAAAACCGA | 188 |
| HtpG3 R | ATTTCATCCGGTGTTCGCAT |  |
| P97 F | ATTGGCGTTAAACCACCTC | 183 |
| P97 R | TGCTTCTTCAAATGCTCGTC |  |
| RAD23 F | ACAACCTGAATCGGCTGACC | 140 |
| RAD23 R | AGGCGAAATACGCTTGAACG |  |
| Hsp70 F | CCAATGGCATTCTTAATGTATCT | 165 |
| Hsp70 R | ACACCTTGTCCCTCTGCTTCT |  |
