# Supplementary figures and images for: Preliminary analysis of Psoroptes ovis transcriptome in different developmental stages
Source: Parasit Vectors. 2016 Nov 4;9:570. doi: 10.1186/s13071-016-1856-z (PMC5096302; doi:10.1186/s13071-016-1856-z)

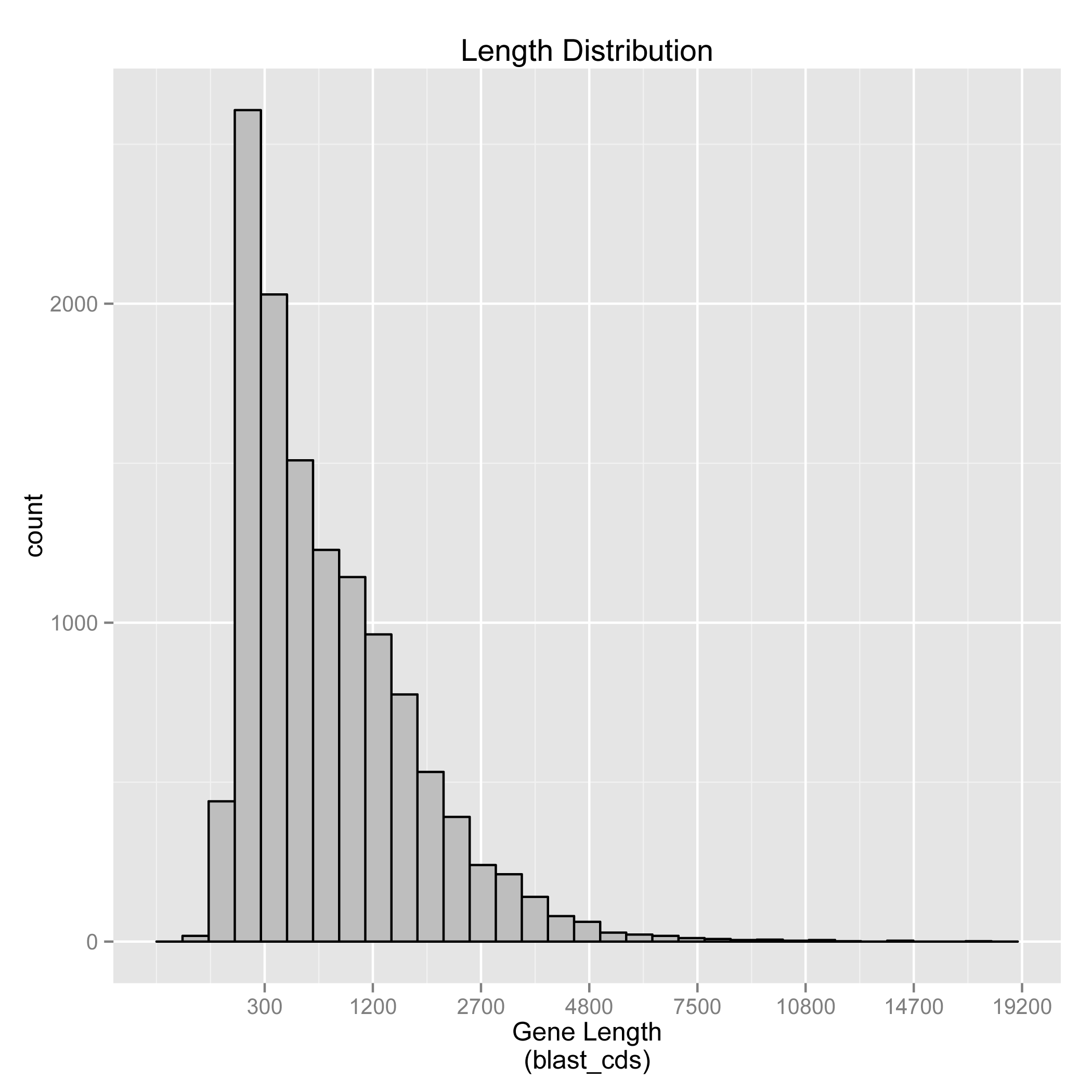

Supplement: Additional file 6: Figure S1. — Length distribution of CDS determined using the Blastx algorithm. (TIF 1334 kb) [file 13071_2016_1856_MOESM6_ESM.tif]

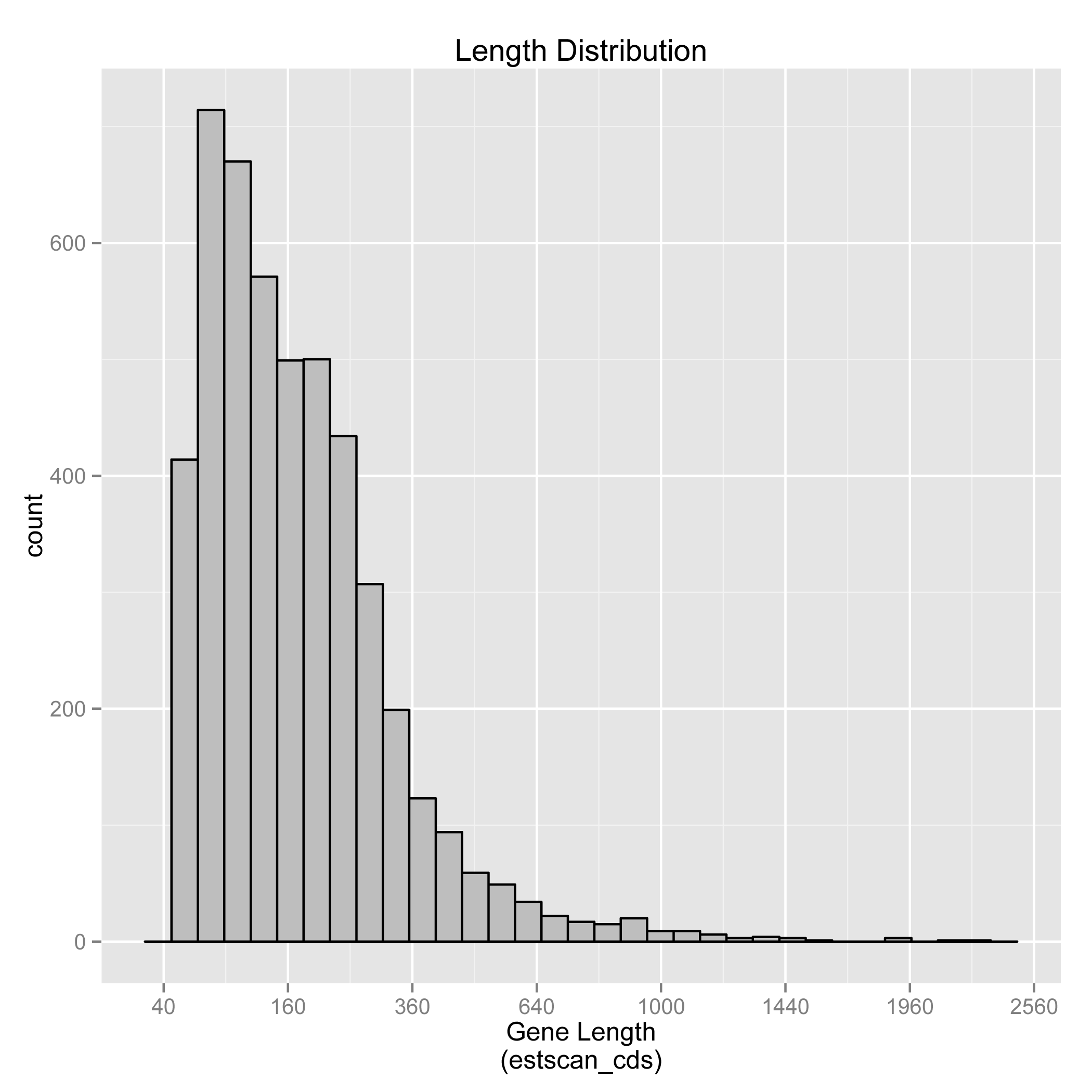

Supplement: Additional file 7: Figure S2. — Length distribution of CDS determined by ESTScan software. (TIF 1342 kb) [file 13071_2016_1856_MOESM7_ESM.tif]

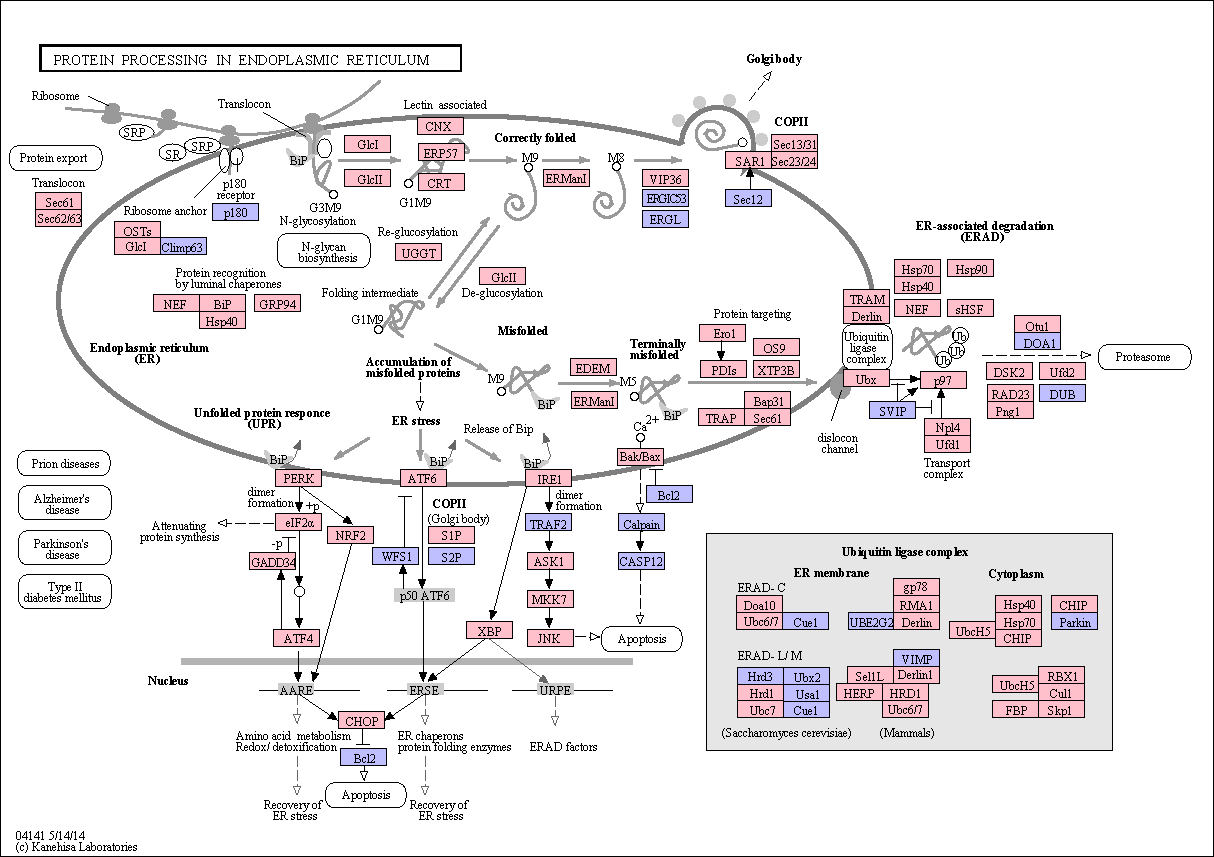

Supplement: Additional file 12: Figure S3. — The ‘Protein processing in endoplasmic reticulum’ pathway. (TIF 46 kb) [file 13071_2016_1856_MOESM12_ESM.tif]
